# *Morinda officinalis* Polysaccharide Supplement Improves Meat Quality in Late-Stage Wenchang Chicken Breeding

**DOI:** 10.3390/biology14091235

**Published:** 2025-09-10

**Authors:** Wei Wu, Hao-Jie Zhang, Quan-Wei Liu, Rui-Ping Sun, Jing-Li Yuan, Yan Zhang, Yu-Hang Liu, Kun Ouyang, Xiu-Ping Wang, Gui-Ping Zhao, Jie Liu, Li-Min Wei

**Affiliations:** 1 Sanya Institute, Hainan Academy of Agricultural Sciences (Hainan Experimental Animal Research Center), Sanya 572025, China; xmxpww@163.com (W.W.); 13250732023@163.com (J.-L.Y.); zhaoguiping@caas.cn (G.-P.Z.); 2 Hainan Key Laboratory of Tropical Animal Breeding and Epidemic Research, Institute of Animal Husbandry & Veterinary Research, Hainan Academy of Agricultural Sciences, Haikou 571100, China; lqw502@126.com (Q.-W.L.); ruiping937@126.com (R.-P.S.); zy79818_0@163.com (Y.Z.); lyh5621744@163.com (Y.-H.L.); 15079542301@163.com (K.O.); 3 College of Animal Science and Technology, Guangxi University, Nanning 530004, China; zhanghj089@126.com; 4 Key Laboratory of Wenchang Chicken Breeding and Feeding, Hainan (Tan Niu) Wenchang Chicken Co., Ltd., Haikou 571100, China; 562856433@163.com

**Keywords:** *Morinda officinalis* polysaccharide, Wenchang chicken, growth performance, meat quality, amino acid contents, fatty acid contents

## Abstract

This experiment aimed to study the effects of adding *Morinda officinalis* polysaccharide (MOP) on the growth, carcass performance and meat quality of Wenchang chickens. A total of 480 eighty-one-day-old Wenchang chickens were randomly allocated into five groups using a completely randomized design, with eight replicates per group (*n* = 8 per treatment). The control group received a basal diet (CON group), while the experimental group was given a diet with 500, 1000, 2000, and 4000 mg/kg MOP supplements. During the 40 day trial, MOP supplementation did not affect the growth and carcass performance (*p* > 0.05), but it increased the a* value of breast muscles compared to the CON group. The breast and thigh muscles in the MOP group had significantly lower shear force than those in the CON group (*p* < 0.05). The MOP groups significantly decreased the muscle fibers’ cross-sectional area and diameter in the breast and thigh muscles of Wenchang chickens compared to the CON group (*p* < 0.05). The MOP_500_ group notably raised the total number of essential amino acids and total amino acid levels in Wenchang chickens, whereas the MOP_1000_ group significantly lowered the total SFA concentration (*p* < 0.05). Collectively, MOP enhances Wenchang chickens’ meat quality, with optimal results at 1000 mg/kg.

## Simple Summary

Currently, there is no research on *Morinda officinalis* polysaccharide (MOP) in Wenchang chickens. However, MOP’s antioxidant properties and potential to enhance meat quality suggest it could be a valuable feed additive. This study establishes that the MOP did not affect the growth and carcass performance. Adding MOP to the diet reduced the shear force and muscle fiber size compared to the control group. The MOP500 group significantly raised total essential and amino acid levels, while the MOP1000 group significantly lowered the total SFA concentration. Overall, MOP enhances Wenchang chicken meat quality, with 1000 mg/kg being the most effective. Using Wenchang chickens and MOP, the research identifies the optimal MOP dosage for these chickens, addressing local production challenges and providing guidance for its use in Hainan.

## 1. Introduction

The Hainan Wenchang chicken, a slow-growing yellow-feathered broiler, is known for its delicious meat and popularity in Southeast Asia. However, its long slaughter age of 120 days and low feed conversion efficiency hinder industry growth.

Hainan Island in China is rich in “southern medicine” resources, like the renowned *Morinda officinalis* (MO), used in traditional Chinese medicine for centuries. MO is one of the four major south medicines. It nourishes the kidneys, strengthens muscles and bones, and relieves rheumatism. *Morindae officinalis* polysaccharides (MOP), extracted from the root of MO, are key active components that mainly comprise rhamnose, arabinose, glucose, and fructose. They combat osteoporosis, boost immunity, and reduce fatigue [1,2]. Huang et al. [3] found that adding MOP boosts antioxidant enzyme activity in chickens with tibial dyschondroplasia; lowers malondialdehyde levels, cooking loss, and shear force after seven days; and reduces oxidative damage in the pectoralis major muscle. Furthermore, MOP has been shown to stimulate the secretion of plasma antioxidant enzymes such as total superoxide dismutase (T-SOD) and glutathione peroxidase (GSH-Px), enhancing the antioxidant capacity of broilers [4]. Kong et al. [5] discovered that 500 mg/kg of MOP lowers serum ALT and AST levels, regulates lipid metabolism, and mitigates intestinal and liver damage in Thiram-exposed chickens. Studies have shown that MOP exhibits an excellent capability to scavenge free radicals and chelate ferrous ions, which are critical mechanisms in reducing oxidative stress [6]. This aligns with findings from other studies, where MOP was found to significantly inhibit oxidative damage in various biological systems, suggesting its broad-spectrum antioxidant potential [7]. The ability of MOP is further supported by its role in improving organismal resistance to oxidative stress, as demonstrated in a study on *Radix Cyathula officinalis Kuan* polysaccharides with similar properties [8].

In addition to its antioxidant properties, MOP has been found to have a range of other beneficial effects, including immunomodulatory and anti-inflammatory activities. These properties contribute to its overall health-promoting effects, which are particularly valuable in the context of animal husbandry, where oxidative stress and inflammation can compromise animal health and productivity [9]. The multifaceted benefits of MOP, including its ability to regulate gut microbiota and enhance bone growth, further underscore its potential as a therapeutic agent in poultry and possibly other livestock [4].

Overall, the evidence supports the notion that MOP is a potent antioxidant agent that can significantly enhance antioxidant enzyme activity in chickens, thereby improving their health and meat quality. This positions MOP as a valuable supplement in poultry nutrition, with potential applications in other areas of animal and human health [3,4,6].

Currently, there is no research on MOP in slow-growing yellow-feathered broilers, such as Wenchang chickens. However, MOP’s antioxidant properties and potential to enhance meat quality suggest it could be a valuable feed additive. This study explores the effects of MOP in broiler diets on growth, carcass traits, and meat quality, aiming to establish it as an effective plant polysaccharide additive. Using Wenchang chickens and MOP, the research identifies the optimal MOP dosage for these chickens, addressing local production challenges and providing guidance for its use in Hainan.

## 2. Materials and Methods

### 2.1. Test Material

The experimental chicken came from Hainan (Tanniu) Wenchang Chicken Co., Ltd. (Haikou, China). The MOP, sourced from the dry roots of *Morinda officinalis* by Shanxi Kangyue Biotechnology Co., Ltd. (Shanxi, China), contained at least 90% polysaccharides (KYBJ230701, phenol-sulfuric acid).

### 2.2. Experimental Design

A total of 480 healthy, 81 day old Wenchang chickens (Tanniu No. 3 breeding line, hens) of similar weight were selected and randomly divided into 5 groups, each with 8 replicates of 12 chickens. The control group received a basal diet, while the experimental group had 500, 1000, 2000, and 4000 mg/kg MOP added to their basal diet. The 40 day feeding trial took place at the Yongfa Experimental Base of the Hainan Academy of Agricultural Sciences’ Institute of Animal Husbandry and Veterinary Medicine. Wenchang chickens were raised in three-layer cages. Chickens had access to ad libitum feed and water. Temperature and humidity were regularly checked during feeding. The basal diet met the nutrient requirements for yellow chickens as per NY/T3645-2020 guidelines. The experimental design and procedures were approved by the Experimental Animal Ethics Committee of Animal Husbandry and Veterinary Medicine Institute, Hainan Academy of Agricultural Sciences (HNSYY20230721). Table 1 displays the nutrient levels and the composition of the basal diet.

### 2.3. Growth Performance

The feed consumption, initial body weight (IBW), and final body weight (FBW) of the chickens during the experiment are recorded in each replicate, and the average daily feed intake (ADFI), average daily gain (ADG), and feed conversion rate (FCR) are calculated. The calculation formula is as follows:

ADG = (FBW − IBW)/Trial days(1)

ADFI = Total feed intake/Trial days(2)

FCR = ADFI/ADG(3)

### 2.4. Carcass Performance

Prior to slaughter, the chickens were fasted for 12 h and then euthanized. The chicken was euthanized by injecting pentobarbital sodium (150 mg/kg) into the wing vein, following the 2020 AVMA Guidelines, and then weighed. A total of 8 chickens randomly selected from each group was weighed for various metrics, including live weight, dressed weight, half-eviscerated weight with giblet, eviscerated weight, breast muscle, thigh muscle, and abdominal fat, following the “Poultry production performance terminology and Measurement Statistical method” (NY/T823/2020). Additionally, percentages for dressing, semi-evisceration, evisceration, breast muscle, thigh muscle, and abdominal fat were calculated.

### 2.5. Sampling Procedure

The breast and thigh muscles from both sides of each carcass were removed, trimmed, weighed, and chilled. The left-side muscles were analyzed for color, pH at 45 min and 24 h, dripping loss, cooking loss, and shear force. The right-side muscles were used to prepare paraffin sections and measure nutritional components, amino acids, and fatty acids.

### 2.6. Meat Quality Determinations

A spectrophotometer (TS7700; Shenzhen 3nh Technology Co., Ltd., Shenzhen, China) was used to measure the meat color values (L*, a*, b*) of each sample three times, and the average was taken [10]. The pH was measured at 45 min and 24 h post-slaughter on the same sections of the breast and thigh muscles using a pH meter (testo 205, Testo Instrument International Trading (Shanghai) Co., Ltd., Shanghai, China).

A 10 g sample of meat was taken from both the breast and thigh muscles. Each piece was weighed, tied with cotton thread, and hung vertically in a cone-shaped bottle, then stored in a 4 °C refrigerator. After 24 h, the meat was reweighed to determine drip loss [11].

To measure cooking loss, breast muscle samples were weighed, sealed in plastic bags, and heated in a water bath to an internal temperature of 80 °C. After heating, the samples were cooled at room temperature for 5 min, dried with absorbent paper, and reweighed to calculate cooking loss [3].

The shear force was determined with a digital muscle tenderness meter (#C-LM3B, College of Engineering, Northeast Agricultural University). After cooking the muscle sample, the shear force was measured at three vertical points along the muscle fibers, and the average of these readings was recorded.

### 2.7. Histology Evaluation

The right breast and leg muscles were harvested and preserved in 4% paraformaldehyde, then processed into paraffin sections [12]. CaseViewer software (2.4.1) captured 10× magnified images of each section, photographing three random locations per section. These images were analyzed with Image J software to measure muscle fiber diameter, number, and total cross-sectional area in three fields of view. Average values were then calculated for muscle fiber diameter, density, and cross-sectional area.

### 2.8. Determination of Nutritional Components

AOAC methods [13] were used for determining moisture content (AOAC 930.15), crude protein content (AOAC 984.13), crude fat content (AOAC 920.39C), and crude fiber content (AOAC 962.09).

### 2.9. Amino Acid and Fatty Acid Contents in Muscle

We referred to Waheed’s method for measuring amino acids in breast muscles [14]. A 50 mg meat sample was hydrolyzed in 6 N hydrochloric acid at 110 °C for 18 h in a sealed container. The hydrolyzed sample was then extracted with citric acid buffer (pH 2.2) and filtered to obtain a clear amino acid solution. Sodium hypochlorite and phthalaldehyde solutions were used for post-column derivatization to determine the amino acid composition of each breast muscle sample.

For fatty acid analysis, following the method of O’Fallon et al. [15], 50 µL of fat or oil was added to a Pyrex nut tube, followed by 1 mL of internal standard, 0.7 mL of KOH solution, and about 5.3 mL of methanol, then mixed. We covered the tube and incubated it in a 55 °C water bath for 90 min. We cooled the tube in water, then added H_2_SO_4_. We placed the tube in a 55 °C water bath for 1.5 h, cooled it, and then added 3 mL of hexane. We mixed the tube in a vortex mixer for 5 min. We isolated and filtered the upper fatty acid methyl ester hexane layer, then analyzed it using gas chromatography. The analysis was performed on a GC-2010 with an FID detector, split flow sampler, and SP-2560 capillary column (100 m * 0.25 mm * 0.2 μm, Supelco, Bellefonte, PA, USA).

### 2.10. Statistical Analysis

Preliminary data processing used SPSS 20.0 (2021). Data are presented as means with a pooled SEM. One-way ANOVA followed by Duncan’s post hoc test was used to examine interactions between multiple variables, and a value of *p* < 0.05 was considered to be a significant difference.

## 3. Results

### 3.1. Effects of MOP on Growth Performance of Wenchang Chickens

As shown in Table 2, dietary supplementation with MOP did not significantly influence growth performance in the 80–120 day Wenchang chickens (*p* > 0.05).

### 3.2. Effects of MOP on Carcass Performance of Wenchang Chickens

As shown in Table 3, dietary supplementation with MOP did not significantly influence carcass performance parameters in Wenchang chickens (*p* > 0.05).

### 3.3. Effects of MOP on Breast Muscle Quality of Wenchang Chickens

Table 4 showed that adding MOP significantly increased the redness (a*) (*p* < 0.05) and decreased the shear force of chest muscles (*p* < 0.01). Furthermore, there were no significant differences in the pH levels, brightness (L*), yellowness (b*), drip loss, or cooking loss among the groups (*p* > 0.05).

### 3.4. Effects of MOP on Thigh Muscle Quality of Wenchang Chickens

Table 5 indicated that the MOP groups had a significant decrease in thigh muscle shear force (*p* < 0.01) compared to the CON group. Additionally, the MOP_1000_ and MOP_2000_ groups showed a significant reduction in thigh muscle drop loss (*p* < 0.05) compared to the CON group.

### 3.5. Effects of MOP on Muscle Histological Characteristics of Wenchang Chickens

**Figure 1:**
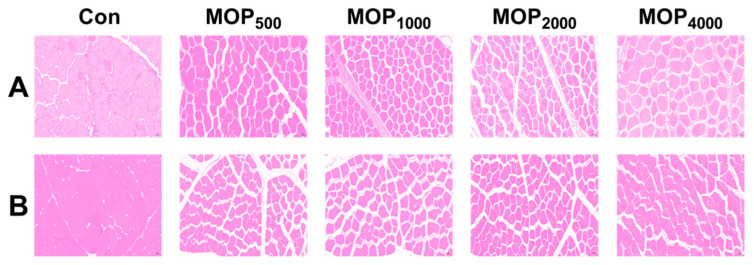
Breast muscle (**A**) and thigh muscle (**B**) histological characteristics, scale bar: 50 μm.

In breast muscle, the MOP_1000_, MOP_2000_, and MOP_4000_ groups displayed smaller cross-sectional areas and muscle fiber diameters but higher muscle fiber density compared to the control (*p* < 0.01), with MOP_1000_ showing the most pronounced effects (*p* < 0.01, Figure 1 and Table 6). Similarly, thigh muscle fibers in MOP groups had smaller cross-sectional areas and diameters, while muscle fiber density increased significantly in the MOP_2000_, and MOP_4000_ groups (*p* < 0.01, Figure 1 and Table 6).

### 3.6. Impact of MOP on Nutrient Content in Wenchang Chicken Breast Muscle

No significant differences were observed in crude fat, crude protein, or moisture content of breast muscles between MOP-supplemented and control groups (*p* > 0.05, Table 7).

### 3.7. Muscle Amino Acid Analysis

As illustrated in Table 8, the MOP_500_ group showed significantly higher levels of total non-essential amino acids (NEAA), and total amino acid (AA) in breast muscle compared to the control and MOP_4000_ groups (*p* < 0.05), with no significant differences observed among the control, MOP_1000_, MOP_2000_, and MOP_4000_ groups.

In the MOP_500_ group, the levels of isoleucine, leucine, glutamic acid, tyrosine, phenylalanine, and glutamate were significantly higher than in the MOP_4000_ group (*p* < 0.05). while other amino acids showed no significant differences among the control and other MOP groups.

### 3.8. Muscle Fatty Acid Analysis

Table 9 indicated that the total saturated fatty acids (SFA) and specific SFA components (e.g., C12:0, C14:0, C15:0, C16:0, and C17:0) decreased significantly in the MOP_1000_ group compared to the control and MOP_500_ group (*p* < 0.05), and with no significant differences among the control and other MOP groups. Additionally, the total polyunsaturated fatty acids (PUFA) and specific PUFA components (e.g., C18:2N6, C18:3N3, C18:1N9C, C18:2N6T, C20:4N6, and C20:2) increased significantly in the MOP_500_ group compared to the MOP_2000_ group (*p* < 0.01).

## 4. Discussion

Currently, there are no known studies on the impact of MOP on broiler growth, although other plant polysaccharides have been researched. For instance, Long et al. [16] found that adding 1 g/kg of *Acanthopanax senticosus* polysaccharides improved ADG and ADFI and reduced FCR in broilers. Yang et al. [17] found that Radix rehmanniae preparata polysaccharides at 600 and 900 mg/kg enhanced body weight gain and FCR in broilers aged 1–35 days. Similarly, Qiao et al. [18] reported that adding *Astragalus membranaceus* or *Glycyrrhiza uralensis* polysaccharides to the diet improved ADG and reduced FCR in broilers aged 1–42 days. *Astragalus membranaceus* polysaccharides and *Glycyrrhiza uralensis* polysaccharides showed growth-promoting effects comparable to antibiotics, suggesting they could replace antibiotics in poultry feed. These studies demonstrate that plant polysaccharides can effectively enhance poultry growth as feed additives. We conducted an initial study on the effects of adding MOP to the feed of broiler chickens, focusing on slow-growing yellow-feathered breeds like the Wenchang chicken. Our study firstly found that adding MOP to diets of the 80 day old Wenchang chickens did not affect the growth performance, unlike the polysaccharide-enriched extract from *Acanthopanax senticosus* [16], *Radix rehmanniae praeparata* [17], and *Astragalus membranaceus* with *Glycyrrhiza* [18]. The possible reason for this is that we use 80 day old Wenchang chickens in the late growth stage, while those studies use one day old chicks.

The experimental results show that adding MOP to broiler feed does not significantly affect carcass performance, possibly due to the type and concentration of the polysaccharides used. pH is a key measure of muscle quality, reflecting the rate of glycogen breakdown in animal carcass post-slaughter. Without aerobic oxidation, glycogen undergoes anaerobic glycolysis, producing lactic acid and releasing H^+^, which lowers the muscle pH [19]. A lower pH reduces meat’s water-holding capacity (WHC), causing PSE meat [20], while a higher muscle pH enhances water retention and extends shelf life [21]. Our research found that adding MOP reduced the pH decrease in breast muscles (*p* = 0.059), with MOP_1000_ being the most effective. Thus, incorporating MOP into the diet can inhibit rancidity and improve broiler chicken quality.

Meat color greatly affects consumer purchasing decisions for livestock and poultry products, with higher a* values being preferred. This study found that MOP notably increased the a* value in breast muscles. Redness in meat can be attributed to an increase in myoglobin, a protein crucial for oxygen storage and transport in muscles [22]. Myoglobin’s heme cofactor binds oxygen, giving muscle its red color. Variations in myoglobin concentration and form, like deoxymyoglobin and oxymyoglobin, alter meat color [23]. Thus, MOP with strong antioxidant properties may maintain the bright red color of muscles by inhibiting myoglobin and lipid oxidation, preventing Fe^2+^ from converting to Fe^3+^, and reducing brown Metmyoglobin formation [24].

Lower dripping and cooking losses indicate better WHC and meat quality. Tenderness, a key quality factor, is measured by shear force; a lower shear force value means greater tenderness [25]. In this experiment, adding MOP to the diet significantly decreased the shear force of breast and thigh muscles. Moreover, 1000 mg/kg and 2000 mg/kg MOP significantly decreased thigh muscle drip loss, with 1000 mg/kg being the most effective. This suggests that MOP can enhance muscle WHC and tenderness. More importantly, 500 mg/kg MOP increased the arginine and proline, suggesting that it enhances meat quality (tenderness, WHC) by boosting metabolites in these pathways [3]. Moreover, MOP enhances meat quality by minimizing oxidative damage and may protect muscle fibers from degradation, potentially improving their quality [3].

The economic value of poultry heavily relies on meat production performance. Muscle fibers, the fundamental components of skeletal muscle, influence poultry meat quality through their physical and chemical properties. The cross-sectional area and diameter of these fibers help assess meat tenderness. Thinner muscle fibers with smaller cross-sectional areas and longer sarcomeres lead to lower shear force, indicating fresher, more tender meat [26,27]. Our study found that adding MOP improves the succulence and tenderness of broiler chicken, boosting its economic value.

Amino acids are vital for meat’s flavor, which greatly influences consumer satisfaction [28]. Leucine supports energy metabolism and protein synthesis while preventing protein breakdown [29]. Isoleucine is also key for protein synthesis [30]. Glycine, alanine, aspartate, glutamate, phenylalanine, and tyrosineare are known as flavor amino acids due to their significant role in enhancing meat’s taste [31]. Incorporating 500 mg/kg of MOP boosts the total NEAA and total AA content in meat (about 82%, and 86%), enhancing the chicken’s nutrition and flavor. However, higher MOP levels reduce amino acid content, indicating that a moderate amount is optimal.

The PUFA/SFA ratio, a key measure of diet’s effect on nutritional value, in chicken usually falls between 0.308 and 2.042 [32]. Our research shows that adding 1000 mg/kg of MOP reduces total SFA concentration (about 20%) in broiler breast meat, without significantly affecting total MUFA and PUFA levels. Higher saturated fatty acid levels can diminish chicken flavor because they oxidize at high temperatures, creating unpleasant-smelling compounds like aldehydes and ketones. These findings indicate that MOP can improve meat flavor and nutrition by changing the fatty acid composition in broiler breast meat, notably reducing C12:0, C14:0, C16:0, C17:0 and C18:0 levels, which are linked to higher serum cholesterol and potential cardiovascular risks. C18:0 seems to have a neutral impact on LDL cholesterol [33]. Our study found that MOP improved meat quality by reducing fatty acids, similar to Guo et al.’s findings with *Chinese Yam* polysaccharide [34].

## 5. Conclusions

This study is the first to show that adding MOP to chicken feed enhances meat quality, notably by improving muscle tenderness and amino acid and fatty acid composition, with the optimal effect at 1000 mg/kg. In summary, MOP is anticipated to be a novel feed additive that supports chicken health, enhances production and meat quality, and boosts economic gains.

## Figures and Tables

**Table 1:** Ingredient composition and nutrient contents of basal diets.

Ingredients, %	Content
Corn	70.50
Soybean meal	17.20
Soybean oil	3.30
Wheat bran	6.00
Limestone	1.20
CaHPO_4_	0.50
NaCl	0.30
Premix ^1^	1.00
Total	100.00
Nutrient levels, %	
Metabolizable energy, MJ/kg	12.82
Crude protein	14.50
Calcium	0.66
Total phosphorus	0.44
Lysine	0.66
Methionine	0.24
Methionine + cysteine	0.51
Arginine	0.88

^1^ The premix provided the following per kg of diets: vitamin A 8.33 KIU, vitamin B_1_ 1.54 mg, vitamin B_2_ 5.21 mg, vitamin B_6_ 2.82 mg, vitamin B_12_ 0.01 mg, vitamin D_3_ 2.08 KIU, vitamin E 41.67 mg, vitamin K_3_ 1.88 mg, biotin 0.13 mg, folic acid 0.54 mg, pantothenic acid 9.38 mg, nicotinic acid 41.67 mg, Cu 5.00 mg, Fe 29.25 mg, Mn 25.00 mg, Zn 25.00 mg, I 0.75 mg, Se 0.23 mg, 99% Met 110.00 g, 78% Lys 285.00 g, and 99% Thr 125.00 g.

**Table 2:** Effects of MOP on growth performance of Wenchang chickens.

Traits ^1^	CON Group	MOP ^2^ Supplemental Levels (mg/kg)				*p*-Value
		500	1000	2000	4000	
IBW (g)	1580.95 ± 1.54	1579.17 ± 2.85	1569.44 ± 4.65	1572.92 ± 4.09	1570.83 ± 2.73	0.075
FBW (g)	2148.61 ± 22.92	2195.24 ± 15.52	2226.04 ± 26.37	2184.17 ± 33.12	2148.61 ± 16.59	0.130
ADG/(g/d)	14.43 ± 0.57	15.33 ± 0.36	16.85 ± 0.64	15.28 ± 0.78	14.87 ± 0.67	0.093
ADFI/(g/d)	94.64 ± 1.09	94.34 ± 1.20	96.67 ± 0.56	92.40 ± 1.38	93.93 ± 1.25	0.143
FCR	6.51 ± 0.24	6.15 ± 0.20	5.87 ± 0.22	5.95 ± 0.22	6.28 ± 0.24	0.284

^1^ IBW, initial body weight; FBW, final body weight; ADG, average daily gain; ADFI, average daily feed intake; FCR, feed conversion ratio. ^2^ MOP, *Morinda officinalis* polysaccharide. Data are presented as the mean ± SEM. The number of samples in each group is eight.

**Table 3:** Effects of MOP on carcass performance of Wenchang chickens.

Traits	CON Group	MOP Supplemental Levels (mg/kg)				*p*-Value
		500	1000	2000	4000	
Dressed percentage (%)	93.89 ± 0.85	94.67 ± 0.66	93.35 ± 0.64	92.93 ± 0.68	93.65 ± 0.78	0.532
Semi-eviscerated percentage (%)	62.22 ± 1.84	64.88 ± 0.88	62.99 ± 0.74	62.75 ± 1.43	63.72 ± 1.52	0.676
Eviscerated percentage (%)	57.42 ± 1.92	60.31 ± 0.90	59.09 ± 0.58	58.96 ± 1.29	60.23 ± 1.16	0.485
Abdominal fat percentage (%)	8.95 ± 0.54	9.68 ± 0.50	9.54 ± 0.78	9.71 ± 0.69	9.31 ± 0.42	0.892
Percentage of breast muscle (%)	16.16 ± 0.50	15.37 ± 0.62	15.25 ± 0.68	15.22 ± 0.59	16.48 ± 0.66	0.472
Percentage of thigh muscle (%)	20.01 ± 1.11	18.66 ± 0.51	20.29 ± 0.48	19.26 ± 0.61	19.80 ± 0.55	0.487

Data are presented as the mean ± SEM. The number of samples in each group is 8.

**Table 4:** Effects of MOP on breast muscle quality of Wenchang chickens.

Traits	CON Group	MOP Supplemental Levels (mg/kg)				*p*-Value
		500	1000	2000	4000	
pH_45min_	5.79 ± 0.08	5.67 ± 0.04	5.72 ± 0.03	5.94 ± 0.11	5.85 ± 0.08	0.115
pH_24h_	5.62 ± 0.02	5.62 ± 0.03	5.69 ± 0.02	5.70 ± 0.03	5.63 ± 0.02	0.059
Brightness (L*)	50.31 ± 0.63	48.99 ± 1.80	48.89 ± 1.16	49.01 ± 0.50	49.66 ± 0.83	0.868
Redness (a*)	−0.29 ± 0.30 ^b^	1.12 ± 0.57 ^ab^	1.43 ± 0.44 ^a^	1.36 ± 0.27 ^ab^	1.59 ± 0.35 ^a^	<0.05
Yellowness (b*)	10.09 ± 0.32	10.41 ± 0.60	9.82 ± 0.92	10.11 ± 0.37	11.24 ± 0.40	0.460
Drip loss (%)	2.20 ± 0.44	2.56 ± 0.42	2.03 ± 0.40	2.36 ± 0.18	2.10 ± 0.43	0.873
Cooking loss (%)	15.22 ± 1.19	20.35 ± 1.87	18.33 ± 0.77	19.65 ± 0.64	19.11 ± 2.44	0.187
Shear force (N)	31.02 ± 2.76 ^a^	22.33 ± 2.44 ^ab^	19.93 ± 1.64 ^b^	21.44 ± 2.11 ^b^	20.14 ± 1.55 ^b^	<0.01

Different superscript letters indicate significant difference (*p* < 0.05). Data are presented as the mean ± SEM. The number of samples in each group is 6.

**Table 5:** Effects of MOP on thigh muscle quality of Wenchang chickens.

Traits	CON Group	MOP Supplemental Levels (mg/kg)				*p*-Value
		500	1000	2000	4000	
pH_45min_	6.15 ± 0.05	6.14 ± 0.01	6.23 ± 0.04	6.22 ± 0.08 ^a^	6.14 ± 0.06	0.590
pH_24h_	5.95 ± 0.03	5.86 ± 0.03	5.88 ± 0.04	5.91 ± 0.03	5.83 ± 0.03	0.118
Brightness (L*)	51.06 ± 0.77	50.49 ± 1.26	50.30 ± 0.81	51.04 ± 0.44	49.39 ± 0.41	0.580
Redness (a*)	7.93 ± 0.63	8.28 ± 0.93	8.68 ± 0.78	7.09 ± 0.53	8.52 ± 0.84	0.601
Yellowness (b*)	16.57 ± 0.55	15.08 ± 0.72	14.23 ± 1.00	15.71 ± 0.79	14.27 ± 0.69	0.183
Drip loss (%)	2.99 ± 0.22 ^a^	2.61 ± 0.17 ^ab^	1.72 ± 0.25 ^c^	2.10 ± 0.21 ^bc^	2.63 ± 0.15 ^ab^	<0.01
Cooking loss (%)	24.36 ± 1.62	21.73 ± 4.31	19.82 ± 3.98	23.07 ± 1.41	23.14 ± 2.14	0.847
Shear force (N)	33.80 ± 2.88 ^a^	24.62 ± 1.69 ^b^	25.01 ± 0.87 ^b^	23.44 ± 1.68 ^b^	24.06 ± 2.04 ^b^	<0.01

Different superscript letters indicate significant difference (*p* < 0.05). Data are presented as the mean ± SEM. The number of samples in each group is six.

**Table 6:** Effects of MOP on muscle histological characteristics of Wenchang chickens.

Traits	CON Group	MOP Supplemental Levels (mg/kg)				*p*-Value
		500	1000	2000	4000	
Breast muscle						
Cross-sectional area (μm^2^)	3628.63 ± 280.96 ^a^	2675.77 ± 177.46 ^b^	1995.47 ± 80.60 ^b^	2369.54 ± 108.79 ^b^	2287.83 ± 157.14 ^b^	<0.01
Diameter (μm)	86.92 ± 3.09 ^a^	75.28 ± 2.57 ^b^	65.78 ± 1.36 ^c^	71.31 ± 1.47 ^bc^	69.75 ± 2.20 ^b^	<0.01
Density (fiber/μm^2)^	290.03 ± 19.77 ^c^	389.59 ± 29.91 ^b^	509.24 ± 20.96 ^a^	430.74 ± 20.75 ^ab^	453.77 ± 28.77 ^ab^	<0.01
Thigh muscle						
Cross-sectional area (μm^2^)	2866.53 ± 107.90 ^a^	2359.09 ± 118.55 ^ab^	2296.65 ± 127.51 ^b^	2018.24 ± 85.41 ^b^	2071.46 ± 197.27 ^b^	<0.01
Diameter (μm)	77.73 ± 1.12 ^a^	70.49 ± 1.50 ^b^	68.92 ± 2.07 ^b^	64.76 ± 1.30 ^b^	64.54 ± 2.35 ^b^	<0.01
Ddensity (fiber/μm^2^)	352.69 ± 12.07 ^b^	433.02 ± 23.84 ^ab^	455.00 ± 36.03 ^ab^	502.89 ± 19.43 ^a^	518.03 ± 43.04 ^a^	<0.01

Different superscript letters indicate significant difference (*p* < 0.05). Data are presented as the mean ± SEM. The number of samples in each group is eight.

**Table 7:** Impact of MOP on nutrient content in Wenchang chicken breast muscle.

Traits	CON Group	MOP Supplemental Levels (mg/kg)				*p*-Value
		500	1000	2000	4000	
Curde fat (%)	7.50 ± 1.38	8.50 ± 1.18	6.33 ± 0.62	5.50 ± 0.56	6.00 ± 1.24	0.275
Curde protein (g/kg)	760.71 ± 14.71	747.82 ± 13.07	779.42 ± 35.4	733.42 ± 39.88	764.72 ± 7.49	0.763
Moisture (%)	68.67 ± 0.33	68.33 ± 0.21	69.20 ± 0.20	69.33 ± 0.33	69.00 ± 0.37	0.135

Data are presented as the mean ± SEM. The number of samples in each group is eight.

**Table 8:** Effects of MOP on the amino acid content of the breast muscle of Wenchang chickens (μg/g).

Traits	CON Group	MOP Supplemental Levels (mg/kg)				*p*-Value
		500	1000	2000	4000	
EAA						
Valine	15.44 ± 3.16	25.96 ± 4.37	20.82 ± 2.59	18.95 ± 4.42	11.19 ± 2.75	0.071
Isoleucine	23.66 ± 5.06 ^ab^	44.7 ± 8.11 ^a^	32.66 ± 4.61 ^ab^	28.74 ± 6.73 ^ab^	14.49 ± 3.02 ^b^	<0.05
Leucine	17.94 ± 3.67 ^ab^	31.51 ± 5.44 ^a^	24.07 ± 3.37 ^ab^	21.31 ± 4.70 ^ab^	11.52 ± 2.31 ^b^	<0.05
Phenylalanine	17.62 ± 3.79 ^ab^	25.64 ± 4.38 ^a^	19.48 ± 2.16 ^ab^	17.07 ± 3.33 ^ab^	10.09 ± 1.62 ^b^	<0.05
Lysine	10.73 ± 2.47	17.40 ± 3.87	14.86 ± 2.39	13.97 ± 3.64	9.93 ± 2.00	0.393
Threonine	18.00 ± 4.15	26.36 ± 3.25	22.31 ± 3.46	17.16 ± 3.06	20.22 ± 5.22	0.482
Methionine	17.37 ± 3.83	25.87 ± 4.37	19.44 ± 2.76	17.23 ± 4.05	10.39 ± 2.15	0.071
Tryptophan	7.22 ± 1.72	10.47 ± 1.67	8.21 ± 1.22	7.73 ± 1.81	4.85 ± 1.31	0.190
NEAA						
Aspartate	21.31 ± 4.94	18.02 ± 3.75	14.12 ± 3.20	18.22 ± 6.28	7.64 ± 1.89	0.231
Arginine	9.24 ± 1.93	13.09 ± 2.17	10.36 ± 1.56	10.30 ± 1.69	5.68 ± 0.59	0.065
Histidine	5.75 ± 1.33	6.52 ± 1.42	6.01 ± 0.79	5.85 ± 0.69	5.74 ± 1.16	0.987
Glycine	4.73 ± 1.21	6.22 ± 1.12	5.54 ± 1.06	4.54 ± 1.18	3.78 ± 0.30	0.517
Alanine	59.30 ± 8.61	70.92 ± 6.32	67.49 ± 5.35	64.57 ± 7.10	57.03 ± 5.26	0.573
Serine	16.90 ± 3.56	22.42 ± 1.13	16.87 ± 1.98	15.68 ± 2.94	11.71 ± 1.79	0.071
Proline	6.44 ± 1.40	8.15 ± 1.01	8.52 ± 1.72	9.20 ± 3.34	6.77 ± 1.50	0.837
Tyrosine	24.07 ± 5.24 ^ab^	34.14 ± 4.97 ^a^	29.32 ± 4.19 ^ab^	21.89 ± 4.76 ^ab^	14.42 ± 2.85 ^b^	<0.05
Cysteine	11.14 ± 1.45	13.22 ± 1.03	12.99 ± 0.61	13.05 ± 1.42	11.12 ± 0.07	0.406
Glutamate	62.04 ± 17.34 ^ab^	120.52 ± 22.44 ^a^	88.53 ± 10.95 ^ab^	74.68 ± 17.3 ^ab^	47.92 ± 9.88 ^b^	<0.05
FAA ^1^	189.08 ± 39.96	275.45 ± 39.91	224.48 ± 23.23	200.96 ± 38.65	152.16 ± 22.25	0.156
Total EAA ^2^	118.05 ± 24.44 ^ab^	207.92 ± 30.01 ^a^	161.85 ± 19.74 ^ab^	142.15 ± 31.10 ^ab^	92.68 ± 18.83 ^b^	<0.05
Total NEAA ^3^	189.71 ± 37.10 ^b^	345.92 ± 30.13 ^a^	254.57 ± 32.36 ^ab^	237.98 ± 44.32 ^ab^	180.15 ± 24.49 ^b^	<0.05
Total AA	307.76 ± 61.24 ^b^	574.97 ± 51.29 ^a^	410.94 ± 54.32 ^ab^	380.13 ± 75.32 ^ab^	283.12 ± 43.48 ^b^	<0.05

^1^ FAA, flavor amino acids; ^2^ EAA, essential amino acids; ^3^ NEAA, nonessential amino acids. Different superscript letters indicate significant difference (*p* < 0.05). Data are presented as the mean ± SEM. The number of samples in each group is six.

**Table 9:** Effect of MOP on fatty acid content of breast muscle of Wenchang chickens (μg/g).

Traits ^1^	CON Group	MOP Supplemental Levels (mg/kg)				*p*-Value
		500	1000	2000	4000	
Total SFA	3239.58 ± 212.03 ^ab^	3414.28 ± 136.48 ^a^	2568.12 ± 70.82 ^c^	2630.44 ± 95.17 ^bc^	2774.79 ± 188.33 ^bc^	<0.01
C8:0	3.49 ± 0.38	3.02 ± 0.28	3.11 ± 0.50	3.01 ± 0.16	3.10 ± 0.23	0.839
C10:0	4.22 ± 0.14 ^ab^	4.48 ± 0.13 ^a^	3.90 ± 0.07 ^b^	4.06 ± 0.06 ^ab^	4.25 ± 0.09 ^ab^	<0.01
C12:0	9.97 ± 0.60 ^a^	10.53 ± 0.62 ^a^	7.78 ± 0.19 ^b^	8.05 ± 0.15 ^b^	9.22 ± 0.32 ^ab^	<0.01
C13:0	4.26 ± 0.12	4.20 ± 0.13	3.33 ± 0.67	2.74 ± 0.87	4.29 ± 0.13	0.134
C14:0	79.42 ± 5.32 ^ab^	84.08 ± 3.76 ^a^	61.67 ± 1.97 ^c^	64.75 ± 1.80 ^bc^	71.42 ± 4.19 ^abc^	<0.01
C15:0	14.03 ± 0.58 ^a^	14.48 ± 0.51 ^a^	11.07 ± 0.49 ^c^	11.48 ± 0.34 ^bc^	13.23 ± 0.62 ^ab^	<0.01
C16:0	2351.67 ± 168.41 ^ab^	2497.70 ± 111.74 ^a^	1816.30 ± 43.69 ^c^	1874.58 ± 68.35 ^bc^	1985.17 ± 150.77 ^bc^	<0.01
C17:0	18.10 ± 0.84 ^ab^	19.66 ± 1.28 ^a^	13.33 ± 0.17 ^c^	13.35 ± 0.40 ^c^	15.79 ± 0.38 ^bc^	<0.01
C18:0	729.25 ± 41.00 ^ab^	757.20 ± 27.78 ^a^	621.92 ± 23.18 ^b^	618.33 ± 23.05 ^b^	647.50 ± 34.76 ^ab^	<0.01
C20:0	11.83 ± 0.26 ^ab^	12.81 ± 0.36 ^a^	10.64 ± 0.27 ^b^	10.90 ± 0.36 ^b^	11.70 ± 0.34 ^ab^	<0.01
C22:0	10.42 ± 0.32 ^a^	4.33 ± 2.76 ^ab^	10.71 ± 0.12 ^a^	9.85 ± 1.98 ^ab^	2.33 ± 2.33 ^b^	<0.01
C24:0	7.96 ± 0.32 ^c^	10.19 ± 0.24 ^a^	8.73 ± 0.32 ^bc^	9.34 ± 0.35 ^abc^	9.93 ± 0.46 ^ab^	<0.01
Total MUFA	301.76 ± 35.06	262.82 ± 30.95	214.50 ± 22.58	230.19 ± 4.20	235.41 ± 34.38	0.230
C16:1	281.08 ± 33.09	242.00 ± 31.40	202.00 ± 19.98	227.00 ± 8.94	220.42 ± 33.65	0.337
C20:1	20.68 ± 2.10 ^a^	20.82 ± 1.51 ^a^	16.03 ± 0.62 ^ab^	14.14 ± 0.50 ^b^	14.99 ± 0.86 ^b^	<0.01
Total PUFA	3646.83 ± 272.95 ^ab^	3927.70 ± 149.14 ^a^	3295.60 ± 83.62 ^ab^	3116.11 ± 91.00 ^b^	3365.81 ± 213.24 ^ab^	<0.05
C18:3N6	15.78 ± 1.07	16.18 ± 0.48	13.41 ± 0.92	13.94 ± 0.85	13.94 ± 1.50	0.237
C18:2N6	1170.25 ± 88.32 ^ab^	1281.17 ± 26.23 ^a^	963.58 ± 24.16 ^b^	962.83 ± 43.31 ^b^	950.50 ± 71.38 ^b^	<0.01
C18:3N3	945.00 ± 64.79 ^ab^	978.30 ± 29.55 ^a^	812.80 ± 16.40 ^ab^	754.58 ± 17.16 ^b^	844.17 ± 74.07 ^ab^	<0.05
C18:1N9C	374.80 ± 24.88 ^a^	392.80 ± 11.90 ^a^	323.10 ± 4.69 ^bc^	306.40 ± 8.19 ^c^	337.50 ± 28.21 ^b^	<0.05
C18:2N6T	164.70 ± 9.08 ^ab^	194.60 ± 13.36 ^a^	143.70 ± 3.12 ^b^	135.58 ± 3.26 ^b^	148.92 ± 11.24 ^b^	<0.01
C18:1N9T	250.83 ± 17.63 ^a^	229.08 ± 15.21 ^ab^	210.70 ± 6.47 ^ab^	195.33 ± 7.14 ^b^	198.83 ± 15.39 ^ab^	<0.05
C20:4N6	656.83 ± 36.76 ^ab^	763.33 ± 108.36 ^a^	531.25 ± 27.62 ^ab^	502.17 ± 33.92 ^b^	579.67 ± 36.06 ^ab^	<0.05
C20:5N3	65.02 ± 3.95	71.75 ± 5.15	69.80 ± 1.93	67.75 ± 2.03	62.30 ± 2.79	0.327
C20:3N6	98.75 ± 6.02	108.00 ± 7.80	94.50 ± 6.06	100.42 ± 2.15	90.75 ± 4.95	0.295
C20:2	53.19 ± 2.31 ^ab^	56.19 ± 2.51 ^a^	48.74 ± 1.26 ^b^	48.90 ± 0.88 ^b^	48.87 ± 0.55 ^b^	<0.05
C22:6N3	118.90 ± 6.52	116.70 ± 14.80	98.40 ± 5.15	87.42 ± 7.71	100.75 ± 6.24	0.091
PUFA/SFA	1.21 ± 0.01	1.19 ± 0.03	1.23 ± 0.03	1.19 ± 0.02	1.23 ± 0.01	0.517
n-6/n-3 PUFA	1.74 ± 0.11	1.78 ± 0.11	1.68 ± 0.05	1.74 ± 0.04	1.65 ± 0.07	0.800

^1^ SFA, saturated fatty acids; MUFA, monounsaturated fatty acids; PUFA, polyunsaturated fatty acids. Different superscript letters indicate significant difference (*p* < 0.05). Data are presented as the mean ± SEM. The number of samples in each group is 6.

## Data Availability

The original contributions generated for this study are included in the article. Further inquiries can be directed to the corresponding author.
